# Bimanual coordination associated with left- and right-hand dominance: testing the limb assignment and limb dominance hypothesis

**DOI:** 10.1007/s00221-021-06082-z

**Published:** 2021-03-22

**Authors:** Stefan Panzer, Deanna Kennedy, Peter Leinen, Christina Pfeifer, Charles Shea

**Affiliations:** 1 Department of Sportscience, Saarland University, Im Stadtwald B8.2, 66041 Saarbrücken, Germany; 2 Department of Health and Kinesiology, Texas A&M University, College Station, TX USA

**Keywords:** Bimanual coordination, Perception–action dynamics, Polyrhythm, Handedness

## Abstract

In an experiment conducted by Kennedy et al. (Exp Brain Res 233:181–195, 2016), dominant right-handed individuals were required to produce a rhythm of isometric forces in a 2:1 or 1:2 bimanual coordination pattern. In the 2:1 pattern, the left limb performed the faster rhythm, while in the 1:2 pattern, the right limb produced the faster pattern. In the 1:2 pattern, interference occurred in the limb which had to produce the slower rhythm of forces. However, in the 2:1 condition, interference occurred in both limbs. The conclusion was that interference was not only influenced by movement frequency, but also influenced by limb dominance. The present experiment was designed to replicate these findings in dynamic bimanual 1:2 and 2:1 tasks where performers had to move one wrist faster than the other, and to determine the influence of limb dominance. Dominant left-handed (*N* = 10; LQ = − 89.81) and dominant right-handed (*N* = 14; LQ = 91.25) participants were required to perform a 2:1 and a 1:2 coordination pattern using Lissajous feedback. The harmonicity value was calculated to quantify the interference in the trial-time series. The analysis demonstrated that regardless of limb dominance, harmonicity was always lower in the slower moving limb than in the faster moving limb. The present results indicated that for dominant left- and dominant right-handers the faster moving limb influenced the slower moving limb. This is in accordance with the assumption that movement frequency has a higher impact on limb control in bimanual 2:1 and 1:2 coordination tasks than handedness.

In bimanual coordination, two limbs perform simultaneously as a synergy. In some situations, the two limbs mutually interact in a way that impedes the individuals’ ability to effectively achieve the goal coordinated bimanual behavior. Sometimes the impediment appears as a performance preference in one limb compared to the other contralateral limb. This phenomenon in bimanual coordination is characterized as performance asymmetry. Welch (1898) was one of the first who described the phenomenon of performance asymmetry in bimanual movements. She reported an experiment where individuals were required to maintain a constant force with one hand while executing a dynamic rhythmical task with the other hand. When the dynamic rhythmical task was assigned to the left hand significantly, greater interference was observed in the right hand producing the constant force (see also Byblow and Goodman 1994). In the years following Welch’s work, researchers in motor control and learning began systematically studying the roles of the two limbs in bimanual coordination in the form of coupling strength and the influence between limbs and performance asymmetries in a variety of bimanual movement coordination tasks (Carson 1989; Haken et al. 1985; Kelso et al. 1979; de Poel et al. 2007; Shea et al. 2016 for a review). For example, Byblow and Goodman (1994) studied a continuous bimanual multi-frequency task, where individuals had to perform a rhythmic movement with a different frequency in each limb simultaneously. Their findings indicated greater stability (less variability) during multi-frequency performance when the right limb of dominant right-handers was assigned to the higher frequency of a rhythmic oscillation task. Peper et al. (1995a) conducted a series of three experiments where skilled drummers were required to perform a bimanual tapping task. They provided empirical evidence that regardless of limb dominance, the faster moving limb was more stable compared to the slower moving limb.

To account for the performance asymmetries in bimanual coordination tasks, a variety of theoretical approaches evolved. Peters (1985) proposed a cognitive related approach in the way that individuals focus more attention on the faster moving hand with interference explained by an attentional bias (Peters and Schwartz 1989). Alternatively, other researchers hypothesized that performance asymmetry was a result of limb assignment. They propose that the faster moving limb has a significant impact on the accuracy and the variability of the slower moving limb (Kennedy et al. 2016; Peper et al. 1995a, b; de Poel et al. 2008; Summers et al. 2002). Another theoretical perspective argues that the asymmetry in performing multi-frequency bimanual movements is due to neural cross-talk (Swinnen 2002; Swinnen and Wenderoth 2004). The basic assumption of the neural cross-talk explanation is that some portion of the signal controlling one hand is also sent as a mirror image of the commands to the homologous muscles of the contralateral limb (Cattaert et al. 1999). Symmetrical iso-frequency bimanual movements are facilitated when contralateral and ipsilateral signals are integrated while multi-frequency bimanual movements suffer from ongoing interference due to conflicting information or partial intermingling of signals controlling the two limbs simultaneously (Cardoso de Oliveira 2002; Kagerer et al. 2003; Marteniuk et al. 1984). Neural cross-talk is also associated with the notion of hemisphere/limb dominance (Kennedy et al. 2016; Serrien et al. 2003). Due to the crossed pathways of the limb neuromotor system for dominant right-handers, the dominant left hemisphere has a greater impact on the non-dominant left hand compared to the non-dominant right hemisphere on the dominant right hand (Haaland and Harrington 1996; Kagerer et al. 2003; Kagerer 2016).

In a recent experiment, Kennedy et al. (2016) contrasted the limb assignment hypothesis and limb dominance hypothesis. They designed an experiment with a Lissajous setting to determine the extent to which the activation of the muscles in one limb influenced the homologous muscles of the contralateral limb during the production of a 1:2 and a 2:1 rhythmical bimanual isometric force production task. Note, the maximum force requirements for both limbs were the same (Kennedy et al. 2017), but in the 1:2 task, the frequency in which the muscle is activated to produce a pattern of isometric forces in the right limb had to be twice as high as in the left limb, while in the 2:1 task, the rhythm of the produced forces of the left limb had to be twice as high as the produced force frequency of the right limb. In a Lissajous setting, integrated concurrent feedback of the produced force pulses of the two limbs was provided and attention demands were reduced (see Shea et al. 2016 for a review). Kennedy et al. demonstrated that in dominant right-handers, distortions in the force time series in a 1:2 bimanual task occurred in the left limb, which produced the lower frequency of force patterns compared to the contralateral right limb, which produced the higher frequency. In the 2:1 condition, distortions in the force time series occurred in the left and right limb. The conclusion was that interference was not only influenced by the increased rhythm of force production (limb assignment) but also affected by limb dominance. However, in terms of limb dominance in the Kennedy et al. (2016) experiment, only dominant right-handers participated. Some research indicated that left limb-dominant individuals do not typically share the same coordination biases as dominant right-handers (Swinnen et al. 1996).

The purpose of the present experiment was to determine the influence of the frequency produced by one limb on the contralateral limb. In the experiment, dominant left-handed and dominant right-handed individuals participated, allowing for a systematic examination of the effects of limb assignment in relation to limb dominance in a multi-frequency bimanual task. Participants were required to rhythmically perform extension flexion movements with the left and right wrists in a 2:1 and 1:2 coordination pattern. Lissajous displays were provided to guide performance and to reduce attentional demands. Note, the experimental focus is primarily on issues related to interference in multi-frequency tasks. For the limb assignment hypothesis, it was predicted that distortions or hesitation in the displacement trace would be observable as non-sinusoidal motion and would primarily occur in the slower moving limb regardless of hand dominance. However, in context of the neural cross-talk approach and the limb dominance hypothesis, it was hypothesized that distortions in the displacement would occur in the non-dominant limb regardless of whether the limb was responsible for the faster or slower frequency.

## Methods

### Participants

Undergraduate students (*N* = 29) volunteered to participate in the experiment after reading and signing a consent form. Handedness was determined by the Edinburgh Handedness Inventory (Oldfield 1971) prior to the experiment. According to a stringent selection criterion of the Laterality Quotient |LQ|> 70, three left-handers and two right-handers were excluded from the experiment. The participants tested were strong right (*N* = 14 dominant right-handed; Oldfield LQ = 91.25) and left (*N* = 10 dominant left-handed; Oldfield LQ = − 89.81) hand dominant (for cutoff definition, see Dragovic 2004). None of the participants was an active musician or had significant training with bimanual movements. Participants received class credit for their participation. The experiment was conducted in accordance laid down in the Declaration of Helsinki (1964). Note, the sample size was estimated a priori by GPower3.1 (Faul et al. 2007; power was 80%, effect size from Kennedy et al. (2016) experiment of *η*_*p*_^*2*^ = 0.30).

### Apparatus

**Fig. 1:**
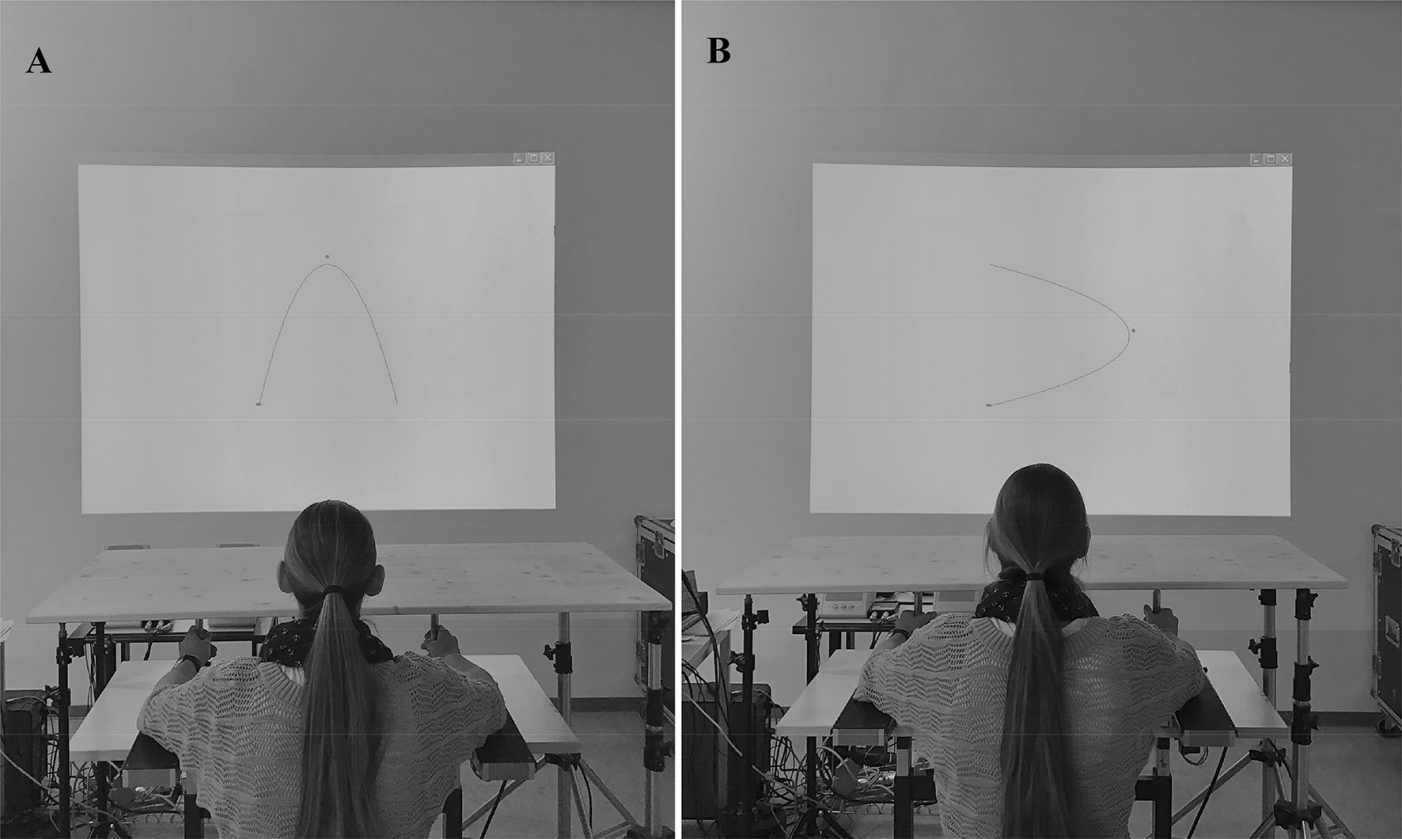
The experimental arrangement with the position of a participant performing the 2:1 multi-frequency task (**a**) and the 1:2 (**b**) multi-frequency task with a Lissajous template

The apparatus consisted of two horizontal levers and a projector. The levers were affixed at the proximal ends to near frictionless axles on the left and right side of the midline of the table. The axles, which rotated freely in ball-bearing supports, allowed the levers to move in the horizontal plane over the table surface (see Fig. 1). Near the distal end of each lever, a vertical handle was attached. The handles’ position was adjusted for shoulder width and hand so that, when grasping the handle with the hand, the participants’ wrist was aligned with the axis of rotation. The hands and the two levers were occluded by a wooden cover placed over the table. A video projector mounted above and behind the participants was used to display the target and a cursor indicating the position of the levers on the wall facing the participant. Participants were seated at about 2 m from the wall and a 1.64 × 1.23 m image was projected onto the wall (see Fig. 1). A potentiometer (Midori, Orange CP-45H 360° endless, resolution < 0.1°) was attached to the lower end of the axis to record the position of the lever and its output voltage was sampled at 200 Hz by a 16-channel A/D converter and stored on a computer for later analysis. The on-line data were used to present a cursor (small red circle) on the wall. The diameter of the projected circle was 2 cm. The projection of the cursor was directly in front of the participant. At the 1:2 and the 2:1 condition, the motion of the left lever moved the cursor up (extension) and down (flexion) and the motion of the right lever moved the cursor left (flexion) and right (extension). The motion of the left and right lever was integrated into a single point in the display. Projected onto the wall was a goal Lissajous template, that represented a 2:1 or a 1:2 pattern of continuous sinusoidal motion (see Fig. 1). The line projected to create the Lissajous template was 1 cm thick. Note, the 2:1 and the 1:2 template differed in that the 1:2 template was rotated 90° clockwise compared to the 2:1 Lissajous template. The Lissajous goal templates were two-dimensional plots that exhibit the desired coordination pattern. The cursor and Lissajous templates were generated with customized software (Matlab 2019a) and displayed with the video projector (1152 × 864 pixel) onto the wall. A height adjustable chair ensured that the participants had sufficiently good visibility of the Lissajous template. Provision of this visual Lissajous feedback occurred in real time. The delay was only limited by the projector refresh rate (100 Hz).

### Tasks and procedure

After entering the testing room, the participant completed the Handedness test and read the written instructions. Then they were positioned at a table with their forearms on a padded aluminum-restraining device fixed on the horizontal support at waist level. This was done to ensure only wrist movements. The participants were instructed to produce rhythmical and continuous flexion–extension movements of the left and the right wrist in a 2:1 or a 1:2 coordination pattern, using the Lissajous display information to guide their performance. The range of motion from the neutral position of the wrist joint was between 10° (dorsal extension) and 35° (palmar flexion). Note that the 2:1 coordination pattern required the participants to move the left wrist twice as fast as the right wrist, while at the 1:2 pattern the right wrist moved twice as fast as the left wrist. The participants were informed that a complete cycle involved extension and flexion of the wrist of about 25°. Following these instructions, the experimenter demonstrated the task one time. Then, left-handed and right-handed participants were required to perform 10 practice trials and 1 test trial for each task. All trials lasted 30 s with a 10 s rest between practice trials and a 5-min rest interval between the practice trials and the test trials. Following the 30 s period, the Lissajous template disappeared. Note, during each trial, individuals repeated multiple times the 1:2 or 2:1 bimanual movement pattern. Between the 2:1 and the 1:2 tasks, there was a 10-min rest interval. The order of the tasks with the different visual displays was counterbalanced. Note, cycle frequency was not guided by a metronome. However, after each practice trial, in which the cycle frequency of the faster moving limb was lower than 1 Hz, the experimenter encouraged the participants to increase their movement speed without disrupting the intended movement pattern.

### Measures and data analysis

Data analysis was performed using MatLab 2019a (MathWorks, Natick, MA, USA). The individual lever displacement time series were used to compute angular velocity and angular acceleration. The data was low-pass filtered with a 2nd-order dual-pass Butterworth filter with a cutoff frequency of 10 Hz.

#### Bimanual performance measure

To examine the ratio of the mean cycle duration, the duration of the fast wrist to the duration of the slow wrist was calculated. A goal ratio of 2.0 would indicate that the interval for the wrist assigned to the faster frequency was twice that of the contralateral wrist. This measure provided temporal information of the goal attainment that is independent of limb coordination tendencies and actual wrist trajectories. Note, a fast to slow ratio was calculated rather than the traditional left limb to right limb ratio. This allows a more direct comparison of the mean cycle duration for the 2:1 and 1:2 tasks.

#### Unimanual performance measures

**Fig. 2:**
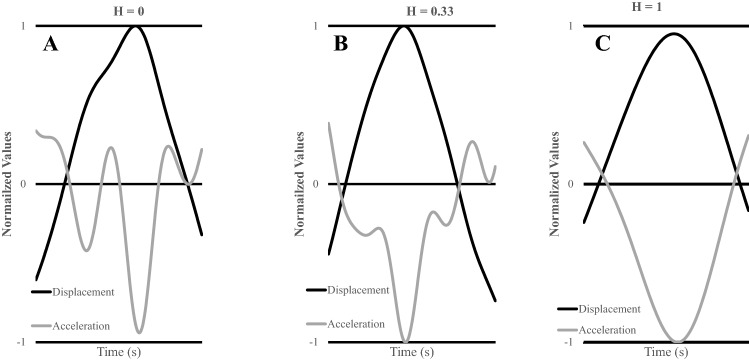
Normalized displacement and acceleration half cycles from one participant showing H = 0 (**a**), H = 0.33 (**b**), and H = 1 (**c**). The subtle changes in the acceleration traces are quantified through the harmonicity analysis. Note, a right-handed participant performed the 2:1 task

To quantify the distortions in the displacement time series the harmonicity value (H) was computed (Kennedy et al. 2015). The H-value quantifies the harmonic or inharmonic nature of the trajectories produced by each wrist for each half-cycle via an analysis of the hesitations in the wrists’ acceleration time series. A 3-point difference algorithm was used to compute the velocity time series and the acceleration time series from the left and right wrists’ displacement time series. The displacement time series for the wrist was mean centered by subtracting out the mean value of the time series from the value itself. This was computed for the left and right wrist. The same procedure was done for the acceleration time series. The displacement and acceleration values were normalized by their maximum values (positive or negative) for each cycle. The normalized displacement and acceleration traces ranged between 1 and − 1. The calculation windows between pairs of zero crossings in the normalized displacement trace were defined to compute H (Guiard 1993). Each non-overlapping time window comprised a single movement reversal. Within each time window, all deflections of the normalized acceleration trace were identified. If the acceleration trace crossed from positive to negative (or vice versa) within this window, the value of H was set to 0, indicating inharmonic motion (see Fig. 2a). When the acceleration trace was positive (negative displacement) within this window, H was computed as the ratio of minimum to maximum acceleration. Conversely, when the acceleration trace was negative (positive displacement) within this time window, H was computed as the absolute ratio of maximum to minimum acceleration (see Fig. 2b). When a single peak (sinusoidal acceleration) occurs in the acceleration trace within this window the value of H was set to 1 (see Fig. 2c), indicating harmonic and continuous motion of the wrist. Finally, the mean of the individual H-values of each time window for a trial was computed as a global estimate of H. A harmonicity value of 1 indicates a harmonic motion of the limbs in which subtle adjustments and/or perturbations are not observable, while an H-value of 0 indicates that the limb motion is inharmonic and one or more adjustments occurred.

Cycle duration and cycle duration variability were computed on a cycle-by-cycle basis with each cycle representing every other zero crossing (Z_Ci_ and Z_Ci+2_). The displacement trace was centered around zero and cycle duration was computed as Z_Ci+2_—Z_Ci_. The cycle duration variability was calculated as the standard deviation of the cycle-to-cycle durations.

#### Statistical analysis

Analyses of variance were computed using the Greenhouse–Geisser corrections when the epsilon value was smaller than 1 (Greenhouse and Geisser 1959). For the bimanual performance measure, a 2 (Handedness: left, right) X 2 (Task: 2:1, 1:2) ANOVA with repeated measures on the last factor was conducted. Unimanual performance measures were analyzed in a 2 (Handedness: left, right) X 2 (Hand: left, right) X 2 (Task: 2:1, 1:2) ANOVA with repeated measures of the last two factors. Partial eta square (*η*_*p*_^2^) was determined as a measure of effect size and was reported for all significant effects (Cohen 1988). Post hoc comparisons of significant interactions were further analyzed with simple main effects analyses using Bonferroni corrections for multiple comparisons when necessary.

## Results

**Fig. 3:**
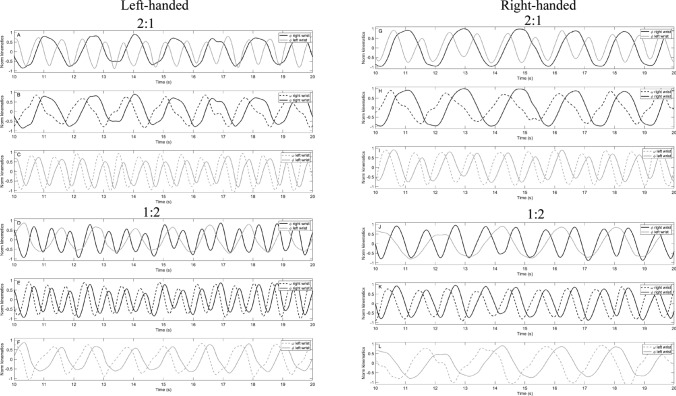
Sample performance of the normalized displacement and normalized velocity time series in the different 2:1 (**a**, **b**, **c**, **g**, **h**, **i**) and 1:2 (**d**, **e**, **f**, **j**, **k**, **l**) tasks for one left-handed (left column) and one right-handed (right column) participant (*ϕ* = angular displacement; *ω* = angular velocity; Norm kinematics = normalized kinematics)

Figure 3 provides a sample of normalized displacement and normalized velocity time series for one participant of each handedness group in the 2:1 and 1:2 conditions.

### Cycle duration ratio

**Fig. 4:**
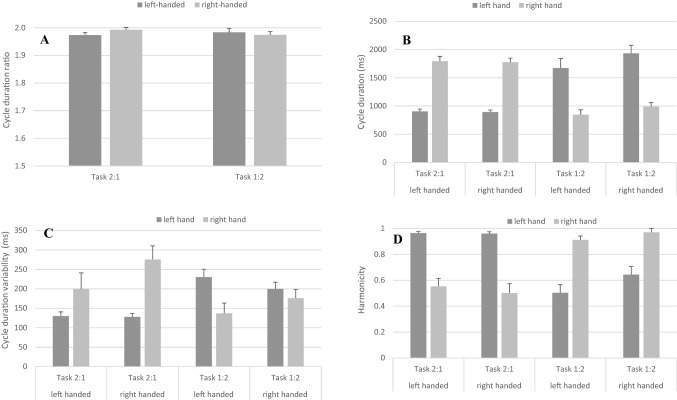
Mean cycle duration ratio (**a**), mean cycle duration (**b**), mean cycle duration variability (**c**) and mean harmonicity (**d**) are provided for each task for left- and right-handed individuals. Error bars represent standard error of the mean

Cycle duration ratios are displayed in Fig. 4a. The analysis of cycle duration ratio indicated no significant main effects of Handedness, *F*(1,22) = 0.21, *p* = 0.65, and Task, *F*(1,22) = 0.14, *p* = 0.71. The interaction Handedness X Task, *F*(1,22) = 1.46, *p* = 0.24, also failed to reach significance.

### Cycle duration and cycle duration variability

Cycle duration and cycle duration variability are displayed in Fig. 4b, c. The analysis of the cycle duration indicated a Task X Hand interaction, *F*(1,22) = 593.74, *p* < 0.01, *η*_*p*_^*2*^ = 0.96. Simple main effect analysis for Task across Hand indicated significantly longer cycle durations for the right wrist compared to the left wrist in the 2:1 task, *F*(1,22) = 948.28, *p* < 0.01, *η*_*p*_^*2*^ = 0.97, and shorter cycle durations for the right wrist compared to the left wrist in the 1:2 task, *F*(1,22) = 270.78, *p* < 0.01, *η*_*p*_^*2*^ = 0.93. The interactions Handedness X Task, *F*(1,22) = 2.25, *p* = 0.15, Handedness X Hand, *F*(1,22) = 1.75, *p* = 0.20, and Handedness X Task X Hand, *F*(1,22) = 0.55, *p* = 0.46, failed to reach significance. In addition, the main effects of Task, *F*(1,22) = 0.07, *p* = 0.78, Hand, *F*(1,22) = 0.01, *p* = 0.99, and Handedness, *F*(1,22) = 0.71, *p* = 0.41, were not significant. This indicated that longer cycle durations were observable when moving the lower than the higher frequency wrist.

However, the analysis of the cycle duration variability indicated a Task X Hand interaction, *F*(1,22) = 80.43, *p* < 0.01, *η*_*p*_^*2*^ = 0.78. The simple main effect analysis for Task across Hand indicated significantly higher cycle duration variability for the right wrist compared to the left wrist in the 2:1 task, *F*(1,22) = 15.59, *p* < 0.01, *η*_*p*_^*2*^ = 0.41, and lower cycle duration variability for the right wrist compared to the left wrist in the 1:2 task, *F*(1,22) = 11.03, *p* < 0.01, *η*_*p*_^*2*^ = 0.34. The other two-way interactions Handedness X Task, *F*(1,22) = 3.65, *p* > 0.05, Handedness X Hand, *F*(1,22) = 3.06, *p* = 0.09, and the three-way interaction Handedness X Task X Hand, *F*(1,22) = 0.05, *p* = 0.82, were not significant. The main effects of Task, *F*(1,22) = 0.09, *p* = 0.77, Hand, *F*(1,22) = 1.41, *p* = 0.25, and Handedness, *F*(1,22) = 0.65, *p* = 0.43, failed to reach significance. As for cycle duration, higher cycle duration variability was shown when moving the lower than the higher frequency wrist.

### Harmonicity

Harmonicity values are displayed in Fig. 4d. The analysis of harmonicity indicated a Task X Hand interaction, *F*(1,22) = 127.88, *p* < 0.01, *η*_*p*_^*2*^ = 0.85. Simple main effect analysis for Task across Hand indicated that the faster moving wrist had higher H-values compared to the slower moving wrist regardless of whether the participant performed the 2:1, *F*(1,22) = 93.28, *p* < 0.01, *η*_*p*_^*2*^ = 0.81, or the 1:2 task, *F*(1,22) = 88.1, *p* < 0.01, *η*_*p*_^*2*^ = 0.80. The interactions Handedness X Task, *F*(1,22) = 4.12, *p* = 0.06, Handedness X Hand, *F*(1,22) = 1.97, *p* = 0.17, and Handedness X Task X Hand, *F*(1,22) = 0.6, *p* = 0.81, failed to reach significance. Furthermore, the main effects of Task, *F*(1,22) = 0.146, *p* > 0.76, Hand, *F*(1,22) = 2.17, *p* = 0.16, and Handedness, *F*(1,22) = 0.53, *p* = 0.48, were not significant.

## Discussion

The primary purpose of the present experiment was to determine the influence of the higher frequency produced by one limb on the contralateral limb performing the lower frequency in dominant right-handed and dominant left-handed individuals. Lissajous feedback was provided as concurrent visual feedback. The experiment was motivated in part from the findings by Kennedy et al. (2016) who reported that the contralateral limb in dominant right-handers was not only influenced by the frequency of the produced forces (limb assignment hypothesis) but also influenced by handedness (limb dominance hypothesis). Further, previous experiments conducted by Amazeen et al. (1997) and Peters (1985) have ascribed the extent to which the faster moving limb influenced the slower moving limb by attentional factors. However, in the current experiment, the two wrists were covered and the perceptual and/or attentional constraints associated with the task were minimized by providing a Lissajous display (see Shea et al. 2016, for a review).

Based upon the cycle duration ratio as a measure of temporal information of the goal attainment in the current experiment, left- and right-handed individuals reached an average cycle duration ratio between 1.94 and 1.99. These ratios are close to the goal ratio of 2. Furthermore, this is confirmed by the individual data of the displacement traces of the left and right limb presented in Fig. 3. In the 2:1 task, the sinusoid of the left wrist showed two cycles while the sinusoid for the right wrist only one cycle. The opposite is observable for the 1:2 task. This indicated that individuals were able to effectively perform the required frequency ratios of 2:1 and 1:2 following 10 trials of practice with Lissajous feedback. This is consistent with previous experiments using a wide range of multi-frequency tasks (e.g., Kennedy et al. 2015; Kovacs et al. 2010; Leinen et al. 2016) and confirms that the Lissajous display with a goal template provided fundamental salient visual information that facilitates the successful performance of a bimanual multi-frequency coordination pattern regardless of whether handedness and whether the limbs were assignment to the 2:1 or the 1:2 coordination pattern. Cycle duration variability was always higher at the slower moving wrist compared to the faster moving wrist. This finding is also consistent with some research on bimanual performance that has shown that performance is less variable when a limb is assigned to the faster frequency (e.g., Byblow and Goodman 1994; Summers et al. 2002) regardless of limb dominance (e.g., Peper et al. 1995a, b). Note, in the three experiments reported by Peper et al. (1995a), the stronger influence of the fast hand on the slow hand was found by skilled drummers with extensive experience in bimanual movements. In the present experiment, the same pattern of result was documented following only 10 trials of practice with Lissajous feedback. Note, the individuals in the current experiment were not active musicians or had significant training with bimanual movements. This finding is in line with previous research (e.g., Kovacs et al. 2010; Leinen et al. 2016; Panzer et al. 2017) that indicates that this form of feedback allowed individuals to effectively perform bimanual coordination patterns following a few minutes of practice, which were once thought to be difficult to perform without extensive practice.

More interesting in the context of the present research and the hypotheses of limb assignment and limb dominance were the harmonicity findings (H-values). Note, the H-value quantifies the distortions in the displacement time series of the left and right wrist when individuals were assigned to the 2:1 or the 1:2 task. The lower H-values in the 1:2 task of the slower moving left wrist were observable in the dominant left- and dominant right-handers. This indicated that distortions in the displacement produced by the slow-moving left wrist could be attributed to the production of the displacement of the faster moving right wrist. Smaller inflections were observed in the right wrist regardless of whether the individuals were dominant left- or dominant right-handers. The same pattern of results occurred for the 2:1 task for dominant left- and dominant right-handers. This pattern of result is consistent with the limb assignment hypothesis which proposed that the faster moving limb has a greater impact on the slower moving limb (Byblow and Goodman 1994; de Poel et al. 2007; Peper and Beek 1998). In the current experiment, an asymmetry in the performance of a 2:1 and 1:2 multi-frequency pattern was observed and that this asymmetry was due to the limb assignment. The statistical analyses are also visualized in the individual examples (see Fig. 3). Additional observations of the individual sample of the displacement traces and the displacement velocity time series for one dominant left- and one dominant right-hander illustrated that there is a tendency that the slower wrist motion tried to wait for the faster wrist motion. At this point of the displacement traces, one wrist was at peak flexion and the other at peak extension. This pattern is observed for left- and right-handed individuals. Regarding the corresponding angular velocity time curve for the slower moving wrist, at this point, the movement was slowed down. It seems that the slower wrist tries to stay in ‘neighborhood’ of the peak positions for a longer time than normally to overcome the inertia of the wrist (see also Peper and Beek 1998; Swinnen et al. 1997).

However, the findings were only partially consistent with previous results provided by Kennedy et al. (2016). The current experiment replicated the results of the Kennedy et al. (2016) experiment for the 1:2 coordination task for the dominant right-handers. However, some of the present findings are contrary to those by Kennedy and colleagues for the 2:1 coordination task. They showed that in dominant right-handers, distortions in a 2:1 task occurred in both limbs, in the dominant right and the non-dominant left limb, whereas in the present experiment interference occurred only in the slower moving wrist. There seem to be some potential reasons that cause the different pattern of result between the previous experiment from Kennedy et al. (2016) and the current study.

An obvious difference between the Kennedy et al. (2016) and the present experiment was that Kennedy and colleagues used a static version of the task which required individuals to perform a 2:1 and a 1:2 multi-frequency force production coordination pattern and in the current experiment, a dynamic version was used. In the static version individuals had to perform an isometric, sequential muscle activation pattern of one muscle group (triceps) with each limb, whereas in the dynamic version in each wrist, a number of flexor muscles (e.g., flexor carpi radialis, flexor carpi ulnaris, palmaris longus, flexor digitorum superficialis, flexor pollicis longus, flexor digitorum profundus) and extensor muscles (e.g., extensor carpi radialis longus, brevis, extensor carpi ulnaris, extensor digitorum, extensor digit minimi, extensor pollicis longus, extensor indicis) were involved and had to be coordinated to produce the task. Furthermore, recent research indicated that proximal effectors induced a different pattern of bimanual performance compared to distal effectors. Research with primates indicated that a large portion of variance in bilateral interference was associated with the interneurons in the spinal cord. The amount of bilateral interneurons connecting muscle groups of the proximal limbs were higher compared to the muscle groups of the distal limbs (Aune et al. 2020). Note, in the Kennedy et al. (2016) experiment, proximal muscle groups were involved while in the current distal muscle groups. Thus, the demands of the task might be a crucial factor in determining the effects of bimanual performance on the costs associated with controlling and switching between different muscle groups (see also Park and Shea 2002), the involved muscle groups, and the anatomical relative locations of the involved limbs.

Another potential reason for the different result pattern might be related to the different sensors in the involved muscles to process proprioceptive information for controlling the bimanual multi-frequency task. According to the previous research on the neuromuscular level, approximately 75% of the afferent muscle spindles increased their discharge and all Golgi tendon organs respond to the force generated at an isometric contraction phase (Edin and Vallbo 1990). Even though, the muscle spindles increased their discharge during an isometric contraction, for the static task the muscle length, predominantly registered by the muscle spindles, is not a crucial factor that had to be controlled during movement execution to reach the goal of the movement. However, for the dynamic task, Golgi tendon organs and the muscle spindles are important sensors to control the continuously changing muscle length during movement execution (Park and Shea 2002). If that information is somehow incorporated into the control of multi-frequency bimanual movements, the between-limb asymmetry would be dependent on the degree to which the information is available or valid during movement execution.

Last, at the isometric bimanual force production task, individuals had to produce different force frequencies between the limbs to achieve goal attainment, while at the dynamic version a different spatial temporal pattern was required between the wrists. Thus, it seems that a different frequency of symmetrical force production between the limbs as in the Kennedy et al. (2016) experiment, the dominant limb exerts a stronger influence on the non-dominant limb. This assumption is in line with previous findings that neural cross-talk is directly related to the strength of innervation or to the actual forces produced by the motor system (Heuer et al. 1998; Walter and Swinnen 1990). Furthermore, at the isometric task the maximum force amplitudes to produce each pattern were similar for both limbs (see Kennedy et al. 2017 for different force amplitudes between the limbs). At the dynamic version of the task, more force is required to produce the faster movement. Therefore, different forces across the limbs are required to perform the dynamic version of the 2:1 or 1:2 coordination patterns. Presumably, different task characteristics seem to accountable that another picture of performance asymmetry in bimanual multi-frequency patterns occurs (see also Armatas and Summers 2001).

The results outlined above hold true for voluntary bimanual movements in healthy participants (e.g., Messier et al. 2006). Research with patients who suffer from hemiplegic neuro motor deficits as a result of stroke indicated that asymmetries between the moving limbs in bimanual tasks occurred while patients focused more on the impaired side whereas healthy individuals concentrate on their preferred limb (Lewis and Byblow 2004). This indicated that movement asymmetries can be altered by hemiplegic motor deficits, and that movement frequency is only one factor that is accompanied by asymmetry in bimanual coordination.

In brief, in a bimanual multi-frequency task where individuals were required to produce different spatial temporal patterns simultaneously with both wrists the distortions occurred predominantly at the slower moving wrist in dominant left- and right-handers. This indicated that the source of interference is primarily due to limb assignment and not to limb dominance. Note, in the current experiment, the moving limbs were covered and Lissajous plots as a source of perceptual information directed the attention toward the movement output and minimized the attentional control of the moving limbs. This changes the way in which movements are controlled, because individuals do not have to split attention between the direct vision of the limbs and the produced effects by the limbs. This is consistent with research of internal and external focus of attention which provided strong evidence that the tendency to control the limb motion has a negative impact on performance (Shea et al. 2016; Wulf 2007 for reviews).

Indeed, theoretical perspectives on bimanual coordination such as the HKB-model (Haken et al. 1985) propose speed is a crucial control parameter that forces the motor system into a different state. Further research can determine the stability of the pattern in the current experiment that the slower limb is affected by the faster limb by increasing the speed (see Bogacz 2005; Treffner and Turvey 1993). Another subject for further research to determine the effect of asymmetry in bimanual control should be to examine the impact of involuntary muscle co-activation with one body side when the contralateral side has to perform an intended movement (Brun et al. 2015). How large the portion of variance of involuntary muscle co-activation is associated with activation of the muscles in the contralateral limb can be observed in involuntary electromyography (EMG). Extending the previous experiment conducted by Kennedy et al. (2016) by the factor hand dominance, our results with strong left- and right-handers suggest that different movement frequencies in a dynamical bimanual multi-frequency movement task haves a higher impact on performance asymmetry than handedness.
